# Interleukin-15 and innate effector cells as predictors of outcome in allogeneic hematopoietic cell transplantation

**DOI:** 10.3389/fimmu.2025.1649313

**Published:** 2026-02-02

**Authors:** Marie Warny, Sisse Rye Ostrowski, Søren Lykke Petersen, Lone Smidstrup Friis, Brian Thomas Kornblit, Niels Smedegaard Andersen, Ida Schjødt, Margit Hørup Larsen, Janne Amstrup Møller, Eva Kannik Haastrup, Henrik Sengeløv, Lia Minculescu

**Affiliations:** 1 Department of Hematology, Danish Red Blood Cell Center, Copenhagen University Hospital - Rigshospitalet, Copenhagen, Denmark; 2 Department of Clinical Immunology, Copenhagen University Hospital - Rigshospitalet, Copenhagen, Denmark; 3 Department of Clinical Medicine, Faculty of Health and Medical Sciences, University of Copenhagen, Copenhagen, Denmark; 4 Department of Hematology, Copenhagen University Hospital - Rigshospitalet, Copenhagen, Denmark; 5 Translational Research, Novo Nordisk A/S, Måløv, Denmark

**Keywords:** cytokines, natural killer cells, gamma delta T cells, allogeneic stem cell transplantation, innate immunology

## Abstract

**Introduction:**

Immune reconstitution is a critical parameter in successful hematopoietic cell transplantation (HCT) and involves different cell types and a microenvironment including cytokines. Natural killer (NK) cells and γδ T cells are known to repopulate early after HCT and are proposed to have the intriguing capacity of mediating graft-versus-leukemia (GVL) effects without accompanying graft-versus-host-disease (GVHD). Interleukin-15 (IL-15) and interleukin-7 (IL-7) are key homeostatic cytokines, with effects on both T and NK cells, making these cytokines especially interesting in an HCT setting.

**Methods:**

In this prospective study, we investigated associations between IL-15 and IL-7, NK cells and γδ T cells, including activated subtypes, and clinical outcomes. We included 105 patients undergoing allogeneic HCT at a single-center institution. IL-15, IL-7, and extended T and NK cell phenotyping were measured longitudinally at fixed time points following HCT.

**Results:**

We found high IL-15 concentrations early post-transplant to be significantly associated with reduced overall survival, reduced relapse-free survival, and excess acute GVHD. Furthermore, IL-15 showed significant inverse correlations with NK cells and γδ T cells, including activated subtypes early after HCT, and with conventional T cells at later time points. IL-7 was significantly inversely correlated not only with conventional T cells but also with γδ T cells early after HCT.

**Discussion:**

These findings may suggest that early immune reconstitution of NK cells and γδ T cells is influenced by the bioavailability of IL-15 after HCT and that IL-15 could have a mechanistic effect in the activity of these innate effector cells. NK cells and γδ T cells are currently being investigated in several promising treatment settings, and IL-15 here may offer a potential benefit.

## Introduction

Hematopoietic cell transplantation (HCT) is a potential curative treatment for malignant hematological disease (1, 2). Even though outcomes have improved drastically during the last decades, approximately 30% experience relapse of the disease (3), and the procedure itself is associated with a high risk of transplant-related mortality (TRM), including graft-versus-host-disease (GVHD), and severe infections post-transplant. The curative principle of allogeneic HCT is the graft-versus-leukemia (GVL) effect, mediated primarily by donor T cells (4). These alloreactive donor T cells detect differences in human leukocyte antigens (HLAs) and minor antigens, expressed on both malignant and non-malignant recipient cells, with allorecognition resulting in GVL and GVHD, respectively (5). In this setting, the overall aim is to identify mechanisms separating GVL and GVHD.

The GVL effect is originally thought to be mediated through conventional αβ T cells, which rely on antigen presentation by classical HLA molecules, with the potential to also induce GVHD. In recent years, however, attention has been drawn towards innate effector cells, primarily natural killer (NK) cells and γδ T cells, both major players in cancer immune surveillance (6–8). They recognize pathogens and cancer cells mainly in an HLA-unrestricted manner (9, 10), implying their sensing of stress signatures to depend on more common changes observed across many individuals (11–15).

NK cells comprise 5%–15% of circulating lymphocytes and are the first lymphocyte population to reconstitute after HCT (16, 17). They exhibit various expression of the neural cell adhesion molecule (NCAM), CD56, inducing functional and phenotypic differences (18). It is suggested that CD56^bright^ NK cells represent precursors of CD56^dim^ NK cells (19). CD56^bright^ NK cells have the capacity for expansion and high-level production of cytokines, stressing a potential immunomodulatory role (20, 21). CD56^dim^ NK cells account for most of peripheral blood NK cells; they prioritize activating and inhibitory receptor input, and have high cytotoxic properties (20, 21).

γδ T cells constitute 1%–10% of circulating T lymphocytes, with several distinct subtypes identified. The subtype Vδ2 is the most prevalent in peripheral blood, whereas Vδ1 resides primarily in tissues, representing approximately 50% of local T cells (22, 23). γδ T cells share features of both the innate and adaptive immune system. They have the capacity to exert direct cytotoxicity, act as antigen-presenting cells (APCs), collaborate with dendritic cells, and enhance anti-infectious activities of NK cells and macrophages, and thereby orchestrate immune responses in combating infections and malignancy (9, 23, 24). Besides the T-cell receptor (TCR) and toll-like receptors, γδ T cells express a wide range of receptors shared with NK cells, such as the activating receptor NKG2D, that engage MCH class I-related molecules (22).

Immune reconstitution after HCT includes development and maturation of a new donor-derived immune system in the recipient. It is an intricate process that includes various cell types and a microenvironment rich in cytokines (25). Cytokines act primarily by binding to receptors on target cells, whereby the cytokine is consumed (26). It is well established that interleukin-7 (IL-7) affects T-cell lymphopoiesis (especially of αβ T cells) and homeostatic peripheral expansion of T cells (27). In line with this, several previous studies have shown an inverse relationship between IL-7 and T-cell count, probably due to a high rate of consumption by T cells (28–31). Interleukin-15 (IL-15) is the main cytokine required for NK cell development, proliferation, homeostasis, function, and survival (32–34). Furthermore, IL-15 plays an important role in γδ T-cell function (35, 36). Despite this, an assessment of the relationship between these two cytokines and specific NK cell subtypes and γδ T cells in a HCT setting is lacking. Previous studies from our group and others have shown that high concentrations of innate effector cells during early immune reconstitution after HCT is associated with improved outcomes (37–45).

The aims of this study are to characterize the cytokine environment during early immune reconstitution and to investigate correlations between cytokine concentrations and innate effector cells in terms of γδ T cells and NK cells. We present data from 105 patients receiving a T-cell-replete stem cell graft. In this population, we previously found early robust reconstitution of NK and γδ T cells to be associated with improved outcome. As we expect high consumption of cytokines during cell expansion after HCT, we hypothesize that IL-15/IL-7 concentrations in the early post-transplant phase may predict outcomes after HCT.

## Materials and methods

### Patients

As previously described in detail (38), 108 patients transplanted at the Stem Cell Transplantation Unit, Department of Hematology, Copenhagen University Hospital, Rigshospitalet from October 2015 to March 2017 were included in the study. The Danish National Committee on Health Research Ethics approved the study, and all participants gave written informed consent prior to transplantation, in accordance with the Declaration of Helsinki. Cytokine measurements were available for 105 of the 108 patients on day 28 after HCT (see **Table 1** for transplant characteristics).

**Table 1:** Patient and transplant characteristics.

N	105
Follow-up time, days, median, range	672 (386–913)
Age, years, median, range	58 (20–74)
Disease, *n* (%)	
AML	45 (43%)
ALL	14 (13%)
MDS	23 (22%)
Myelofibrosis	8 (8%)
Lymphoma	6 (6%)
Chronic leukemia	4 (4%)
Other	5 (5%)
DRI, *n* (%)	
Low	62 (56%)
Intermediate	42 (39%)
High	5 (5%)
Graft source, *n* (%)	
BM	15 (14%)
PBSC	90 (86%)
Donor, *n* (%)	
MRD	23 (22%)
MUD	80 (76%)
Haploidentical	2 (2%)
Donor match, *n* (%)	
10/10 or 9/10 allele match	94 (90%)
1 Ag MM	9 (8%)
Haploidentical	2 (2%)
Recipient–donor sex, *n* (%)	
M/M	51 (49%)
M/F	27 (26%)
F/F	16 (15%)
F/M	11 (10%)
Conditioning intensity, *n* (%)	
Myeloablative	48 (46%)
Non-myeloablative	57 (54%)
Conditioning regimen, *n* (%)	
TBI-Flu	53 (50%)
Flu-Treo	24 (23%)
TBI-Cy	21 (20%)
TBI-Etopophos	4 (4%)
Other	3 (3%)

AML, acute myeloid leukemia; MDS, myelodysplastic syndrome; ALL, acute lymphoblastic leukemia; DRI, Disease Risk Index; BM, bone marrow; PBSC, peripheral blood stem cell; MDR, matched related donor; MUD, matched unrelated donor; TBI, total body irradiation; Flu, fludarabine; Treo, treosulfan; Cy, cyclophosphamide.

### Transplant procedures and definitions

Transplant procedures have previously been described in detail (38). Briefly, myeloablative conditioning was cyclophosphamide 120 mg/kg (Etophophos 1,800 mg/m^2^ for ALL) and 12 Gy total body irradiation (TBI) or fludarabine 150 mg/kg and treosulfan 42 g/m^2^ in patients with myelodysplastic syndrome (MDS). Non-myeloablative conditioning was fludarabine 90 mg/m^2^ and 2–4 Gy TBI. Patients receiving a haploidentical HCT were conditioned with cyclophosphamide 29 mg/kg, fludarabine 150 mg/m^2^, and 2 Gy TBI.

Twelve patients received anti-thymocyte globulin (ATG) as part of their conditioning regimen: two patients with antigen-mismatch received thymoglobulin 2.5 mg/kg and 10 patients with matched unrelated donors, transplanted with peripheral blood stem cells (PBSCs), received Grafalon 10 mg/kg.

GVHD prophylaxis in myeloablative regimens included cyclosporin and short-course intravenous methotrexate on days 1, 3, 6, and 11. Cyclosporine was tapered to stop day 180 unless GVHD was present. In non-myeloablative regimens, tacrolimus and mycophenolate mofetil were administered; tacrolimus was tapered from day 56 to 180 in related transplants, and from day 100 to 180 in unrelated transplants, in the absence of GVHD. Tacrolimus was administered in the fludarabine/treosulfan regimen. Mismatched non-myeloablative transplant patients were treated with cyclosporine, sirolimus, and mycophenolate mofetil, tapered to stop days 180, 365, and 150, respectively, in the absence of GVHD. In haploidentical transplants, cyclophosphamide 50 mg/kg (days 3 and 4), tacrolimus, and mycophenolate mofetil were administered.

Acute GVHD (aGVHD) was diagnosed and graded from clinical symptoms and biopsies, according to the modified Glucksberg–Seattle criteria (46, 47).

In leukemia patients, relapse was defined as more than 5% blasts in the bone marrow, or the appearance of extramedullary leukemic lesions. In MDS, relapse was defined as recurrence of MDS by morphology, cytogenetics, or both. In lymphoma, relapse was defined as new or progressing foci on positron emission tomography/computed tomography (PET/CT) scans.

### Patient samples

Blood samples were collected at five time points (median): day 28 (range, 23–39), day 56 (48–76), day 91 (74–122), day 180 (148–239), and day 365 (334–452) after HCT. Samples were analyzed freshly by flow cytometry at the Tissue Typing Laboratory, Department of Clinical Immunology, Copenhagen University Hospital, Rigshospitalet. Plasma for cytokine analyses was extracted from each sample and frozen at −80°C.

### Cytokine assay

To quantify the concentrations of IL-7 and IL-15 in patient plasma samples, specific immunoassay techniques were employed at the Department of Clinical Immunology, Copenhagen University Hospital, Rigshospitalet. Briefly, for both assays, plasma samples including internal plasma controls were thawed and vortexed and then aliquoted to assay plates using a Hamilton Star Liquid handler, reducing the variability and bias due to manual pipetting.

Concentrations of IL-7 were determined using an enzyme-linked immunosorbent assay (ELISA) platform. The ELISA assays were performed using commercial IL-7 kits following the manufacturer’s protocol (Quantikine^®^ HS ELISA, Cat. No. HS750, R&D Systems Europe, Ltd.). All samples were diluted fourfold with diluent buffer. The optical density was measured at the recommended wavelength, and concentrations were calculated based on the standard curve generated with known IL-7 concentrations.

IL-15 concentrations were measured using Luminex^®^ technology. Following the manufacturer’s protocol, the Luminex assays were performed using a Luminex^®^ Discovery Assay Kit (Cat. No. LXSAHM, R&D Systems Europe, Ltd.). All samples were diluted twofold. Data were acquired and analyzed using specialized software, with concentrations determined by referencing a standard curve.

Internal controls were included in duplicates in each run for both ELISA and Luminex measurements to ensure accuracy and consistency across all measurements. Intra- and inter-assay coefficients of variation were determined to be <20%.

### Flow cytometry and lymphocyte phenotyping

As previously described (38, 39), a multi-color flow cytometry panel including the following antibody-staining combination was used: CD3, TCRαβ, TCRγδ, TCRVδ1, TCRVδ2, HLA-DR, CD16, CD56, and CD314 (NKG2D) (see **Supplementary Table S1**). Gating strategies have previously been described (38, 39) and are also presented in **Supplementary Figure S1**. Analyzed lymphocyte subsets were absolute concentrations of CD3 T cells, αβ T cells, γδ T cells, Vδ1 T cells, Vδ2 T cells, CD4 T cells, CD8 T cells, total NK cells, CD56^dim^ NK cells, and CD56^bright^ NK cells. The expression of HLA-DR as a marker of activation was analyzed on T cells, and the expression of the activating receptor NKG2D was analyzed on γδ T cells and NK cells. For subset biomarkers, see **Supplementary Table S2** and (38, 39). For this study, only selected subtypes were used for analyses.

### Outcomes

The primary outcomes were overall survival (OS), relapse-free survival (RFS), and aGVHD from day 28 after HCT. OS was defined as the probability of survival from day 28 with death as the event. RFS was defined as the probability of survival without relapse from day 28 with an event defined as the composite of relapse or death. Risk of aGVHD was defined as risk of developing aGVHD grade II–IV from day 28. Seven patients were diagnosed with aGVHD before their respective day 28 sample and were therefore excluded from the aGVHD analyses. Day 28 after HCT was selected for the primary outcome, as this was the closest time point to stem cell infusion, and therefore the most interesting when considering the cytokine environment.

### Statistical analyses

Differences in plasma concentrations of IL-15 and IL-7 during the first year after HCT were assessed using linear mixed models. Kaplan–Meier survival analysis and Cox proportional hazard regression models were used to investigate the associations between cytokine concentrations and OS and RFS. Based on Cox models, we also calculated risk estimates according to continuous levels of cytokines using restricted cubic splines, presented with four knots, chosen based on Akaike’s information criteria (48). In addition to cytokine concentrations, pre-transplant factors thought to have a possible impact on the primary endpoints were included in the analysis. Disease Risk Index was included for all patients after previously published criteria (49). Pre-transplant factors were analyzed in patients alive by day 28 after HCT with available cytokine measurements (*n* = 105), and variables known to be associated with death, relapse, and aGVHD were included in multivariable adjusted models and included donor age (above vs. below age 30), HLA match (other vs. 9–9/10), ATG use (vs. no ATG use), donor type (MUD vs. MRD), sex mismatch (female–male vs. other), and conditioning regimen [myeloablative (MA) vs. non-myeloablative (NMA)]. The cumulative incidence of aGVHD and relapse-related mortality (RRM) was determined using Fine&Gray’s competing risks analysis. Competing risk for aGVHD was death from all causes other than aGVHD. Competing risk for death from relapse was death from all causes other than relapse (TRM).

Cytokine measurements and cell concentrations were non-normally distributed and therefore analyzed as categorical (dichotomized by the median value) or continuous log-transformed variables in all main analyses. Spearman correlation was used for non-parametric assessment of correlation between different cell subsets and cytokines during immune reconstitution. A *p*-value ≤ 0.05 was considered a statistically significant result.

Statistical analyses were performed using STATA version 18.5, SPSS version 22 (SPSS, Chicago, IL) and R version 3.2.0 (R Foundation for Statistical Computing, Vienna, Austria) combined with the EZR platform (50).

## Results

### Patient outcome

After a median of 672 (386–913) days after HCT, 78 (74%) of the 105 patients were alive; 13 (13%) patients died from TRM, and 14 (13%) died from relapse. A total of 24 (23%) experienced relapse during the follow-up time with a median time to relapse of 177 (56–778) days. aGVHD grade II–IV was diagnosed in 38 (36%) patients.

For results of univariate analyses investigating the association between pre-transplant factors and outcomes, see **Supplementary Table S3**.

### Cytokine concentrations after HCT

**Figure 1:**
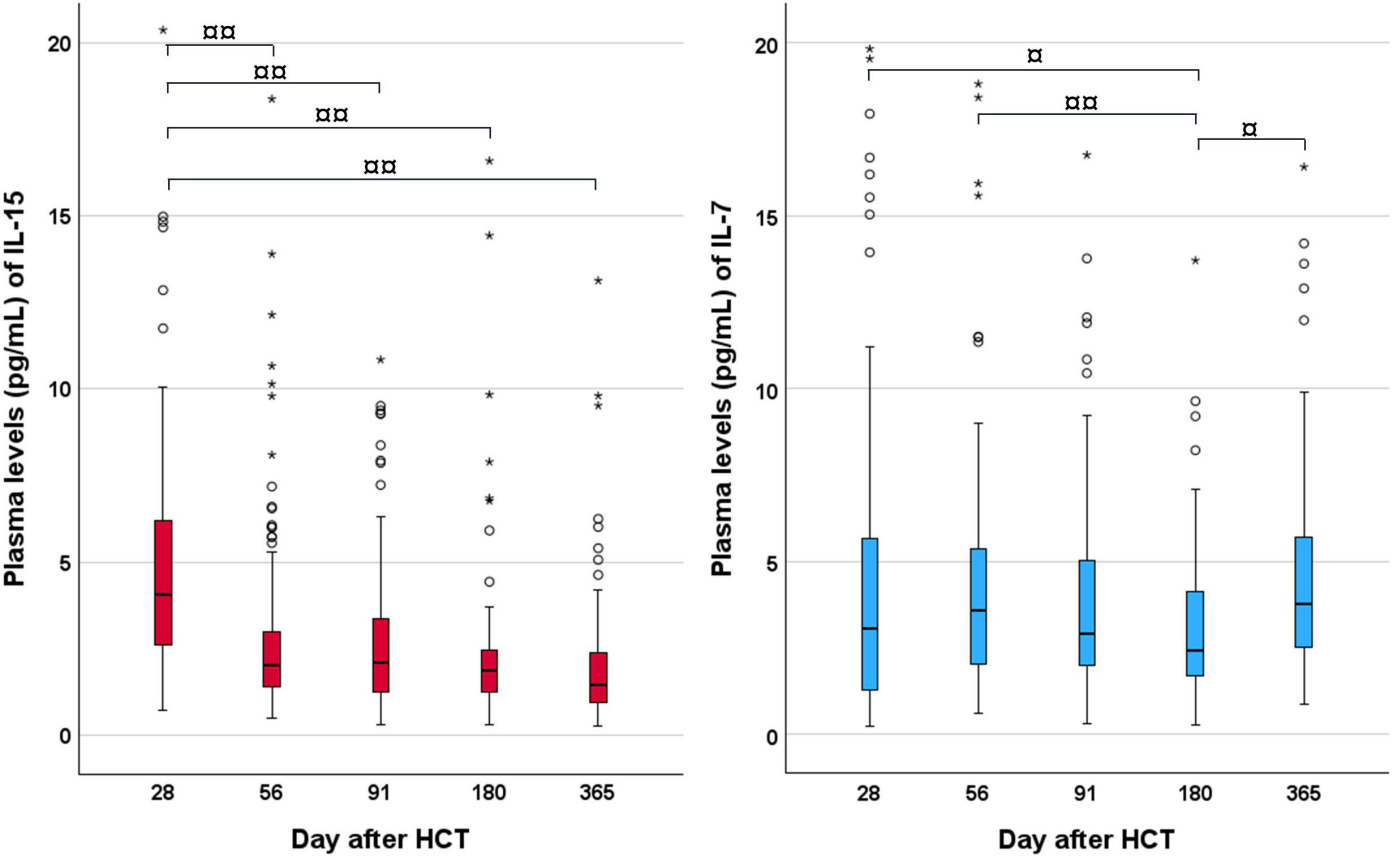
Plasma concentrations of IL-15 (left) and IL-7 (right) during the first year after HCT. Boxes represent the median value and 25th and 75th percentiles. The upper whisker represents 1.5 times the IQR and the lower whisker represents the minimum value. Circles (○) and stars (*) represent outliers. For graphical presentation, a few outliers have been removed: day 28 (four outliers for both IL-15 and IL-7), day 56 (one outlier for IL-15 and 2 for IL-7), day 91 (one outlier for IL-15), and day 365 (one outlier for IL-7). ¤ indicates *p* < 0.05, ¤¤ indicates *p* < 0.001. HCT, hematopoietic cell transplantation. Reprinted with permission from [Improved Overall Survival, Relapse-Free-Survival, and Less Graft-vs.-Host-Disease in Patients With High Immune Reconstitution of TCR Gamma Delta Cells 2 Months After Allogeneic Stem Cell Transplantation] by [Lia Minculescu, Hanne Vibeke Marquart, Lars Peter Ryder, Niels Smedegaard Andersen, Ida Schjoedt, Lone Smidstrup Friis, Brian Thomas Kornblit, Søren Lykke Petersen, Eva Haastrup, Anne Fischer-Nielsen, Joanne Reekie, and Henrik Sengelov], [Frontiers in Immunology].

**Figure 1** and **Supplementary Table S4** show concentrations of IL-15 and IL-7 on days 28, 56, 91, 180, and 365 after HCT. Concentrations of IL-15 were highest on day 28 after HCT and decreased the further away from transplantation. Concentrations of IL-7 showed a more fluctuating tendency. Both cytokines showed the broadest range of concentrations on day 28 after HCT. **Table 2** shows the distribution of pre-transplant factors associated with IL-15 and IL-7 concentrations 28 days after HCT. Patients receiving ATG had a significantly higher IL-7 concentration on day 28 after HCT (*p* = 0.002) and a tendency towards lower concentrations of IL-15 (*p* = 0.06). Furthermore, concentrations of IL-15 were significantly higher in patients receiving bone marrow as their stem cell source (*p* = 0.01).

**Table 2:** Distribution of pre-transplant factors associated with IL-15 and IL-7 concentrations 28 days after HCT.

IL-15	IL-7 high	IL-7 low	*p*-value (χ^2^)
*N*	53	52	
Age			
≤45	9 (38%)	15 (63%)	0.2
>45	44 (54%)	37 (46%)	
DRI			
0–1	51 (54%)	43 (46%)	0.02
2	2 (18%)	9 (82%)	
Donor age			
≤30	30 (50%)	30 (50%)	0.9
>30	23 (51%)	22 (49%)	
Recipient–donor sex			
Female–male	5 (45%)	6 (55%)	0.7
Other	48 (51%)	46 (49%)	
Donor type			
MRD	10 (43%)	13 (57%)	0.3
MUD	41 (51%)	39 (49%)	
Haploidentical	2 (100%)	0 (0%)	
HLA-match			
9/10 or 10/10 allele match	45 (48%)	49 (52%)	0.1
Other (antigen MM + haploidentical)	8 (73%)	3 (27%)	
Stem cell source			
BM	12 (80%)	3 (20%)	0.01
PBSC	41 (46%)	49 (54%)	
Conditioning regimen			
Myeloablative	22 (46%)	26 (54%)	0.4
Non-myeloablative	31 (54%)	26 (46%)	
ATG			
Yes	3 (25%)	9 (75%)	0.06
No	50 (53%)	43 (46%)	

IL-7	IL-7 high	IL-7 low	*p*-value (χ^2^)
*N*	53	52	
Age			
≤45	12 (50%)	12 (50%)	1
>45	41 (51%)	40 (49%)	
DRI			
0–1	50 (53%)	44 (47%)	0.1
2	3 (27%)	8 (73%)	
Donor age			
≤30	26 (43%)	34 (57%)	0.9
>30	27 (60%)	18 (40%)	
Recipient–donor sex			
Female–male	5 (45%)	6 (55%)	0.7
Other	48 (51%)	46 (49%)	
Donor type			
MRD	15 (65%)	8 (35%)	0.1
MUD	36 (45%)	44 (55%)	
Haploidentical	2 (100%)	0 (0%)	
HLA-match			
9/10 or 10/10 allele match	45 (48%)	49 (52%)	0.1
Other (antigen MM + haploidentical)	8 (73%)	3 (27%)	
Stem cell source			
BM	9 (60%)	6 (40%)	0.4
PBSC	44 (49%)	46 (51%)	
Conditioning regimen			
Myeloablative	23 (48%)	25 (52%)	0.6
Non-myeloablative	30 (53%)	27 (47%)	
ATG			
Yes	11 (92%)	1 (8%)	0.002
No	42 (45%)	51 (55%)	

### Cytokine concentrations correlate with innate effector cells 28 days after HCT

#### IL-15

**Figure 2:**
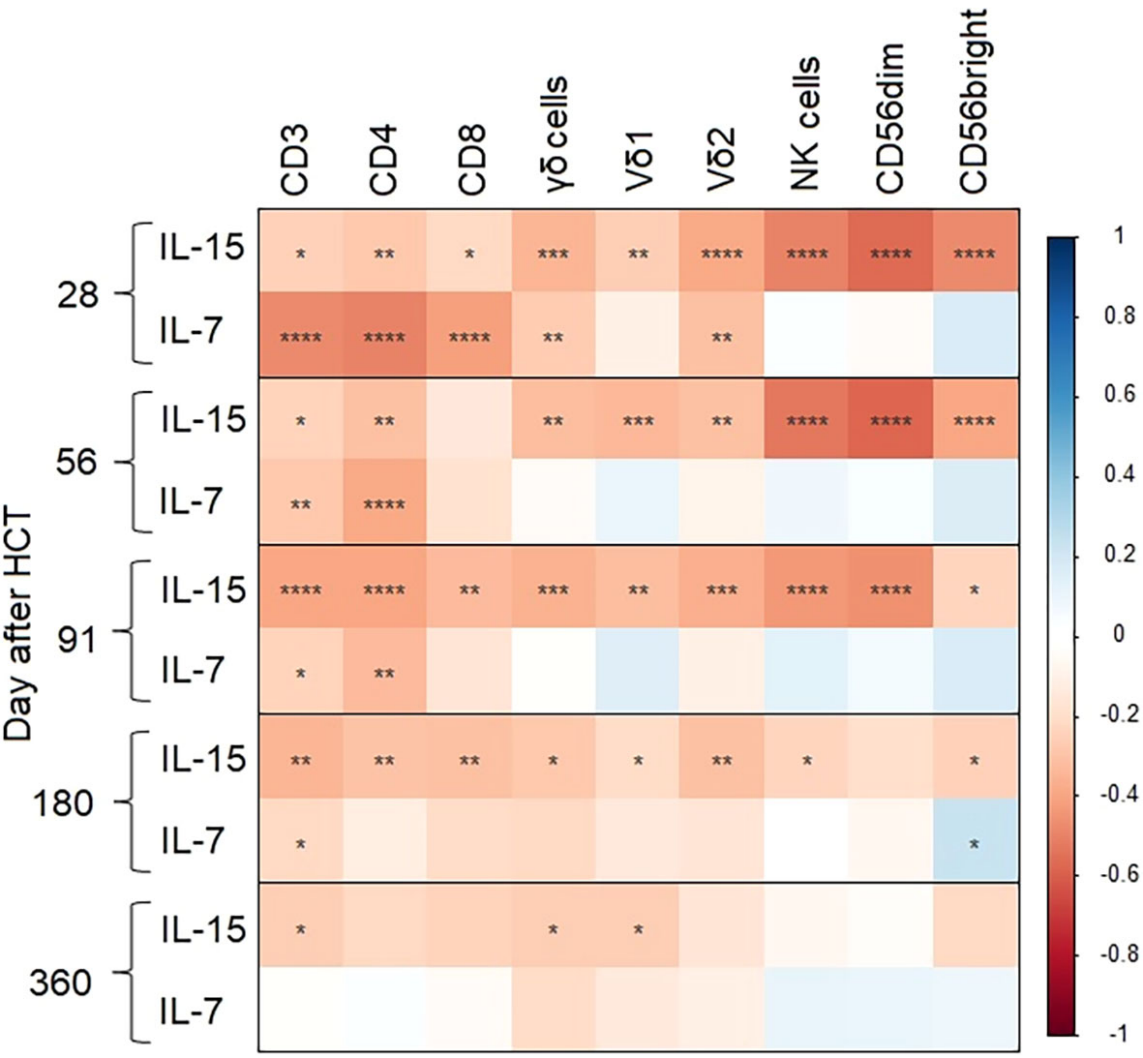
Correlations between cytokines and innate effector cells on days 28, 56, 91, 180, and 365 after HCT. Calculations are based on concentrations of cytokines (IL-15 and IL-7) and innate effector cells (γδ T cells, NK cells, and subtypes) at the indicated time points after HCT. The colors represent the Spearman correlation coefficient at each time point with the corresponding *p*-value (*p < 0.05, **p < 0.01, ***p < 0.001, ****p < 0.0001).

IL-15 was negatively correlated with γδ T cells (*p* < 0.001, **Figure 2**, **Supplementary Table S5**), especially represented by the Vδ2 compartment (*p* < 0.0001), but also the Vδ1 compartment (*p* = 0.01). When investigating the correlation with the activating receptor NKG2D on γδ T cells, the same pattern was observed for all γδ T cells (*p* < 0.001), Vδ2 T cells (*p* < 0.001), and Vδ1 T cells (*p* = 0.05). Furthermore, IL-15 was negatively correlated with the activating receptor HLA-DR on γδ T cells (*p* = 0.002) and with the fraction of γδ T cells of all CD3 cells (*p* = 0.004). All correlations tended to attenuate further away from the transplantation date (**Figure 2**).

Similarly, all correlations between IL-15 and NK cells were inverse and significant (*p* < 0.001) when investigating all NK cells, subgroups of NK cells (CD56^dim^ and CD56^bright^), and the activating receptor NKG2D on NK cells, but with a trend towards non-significant values the further away from the transplantation date (**Figure 2**; **Supplementary Table S5**).

IL-15 was negatively correlated with all T cells (CD3^+^), and with CD4^+^ and CD8^+^ T cells (*p* = 0.01, 0.004, and 0.02, respectively). Interestingly, these correlations tended to strengthen the further away from the transplantation date, with the strongest correlation on day 91 (**Figure 2**; **Supplementary Table S5**).

#### IL-7

IL-7 was negatively correlated with γδ T cells (*p* = 0.006) and with the activating receptor NKG2D on γδ T cells (γδCD314, *p* = 0.02) (**Figure 2**; **Supplementary Table S5**).

IL-7 was negatively correlated with all T cells (CD3^+^), and with CD4^+^ and CD8^+^ T cells (*p* < 0.0001). Furthermore, we found a significant negative correlation with activated CD4^+^ T cells (HLA-DR pos) (*p* < 0.001). The associations between IL7 and γδ T cells, γδCD314, CD3^+^, CD4^+^, CD8^+^, and HLA-DR^+^ CD4^+^ T cells decreased on day 56 after HCT but remained significant for overall T cells and CD4^+^ T cells (**Figure 2**; **Supplementary Table S5**).

We found no negative correlations between IL-7 and NK cells.

Concentrations of IL-7 and IL-15 were not significantly correlated at any time point (data not shown).

For absolute cell concentrations of T cells, NK cells, and subsets in patients and healthy donors, see **Supplementary Table S6**.

### Cytokine concentrations 28 days after HCT and clinical outcomes

**Figure 3:**
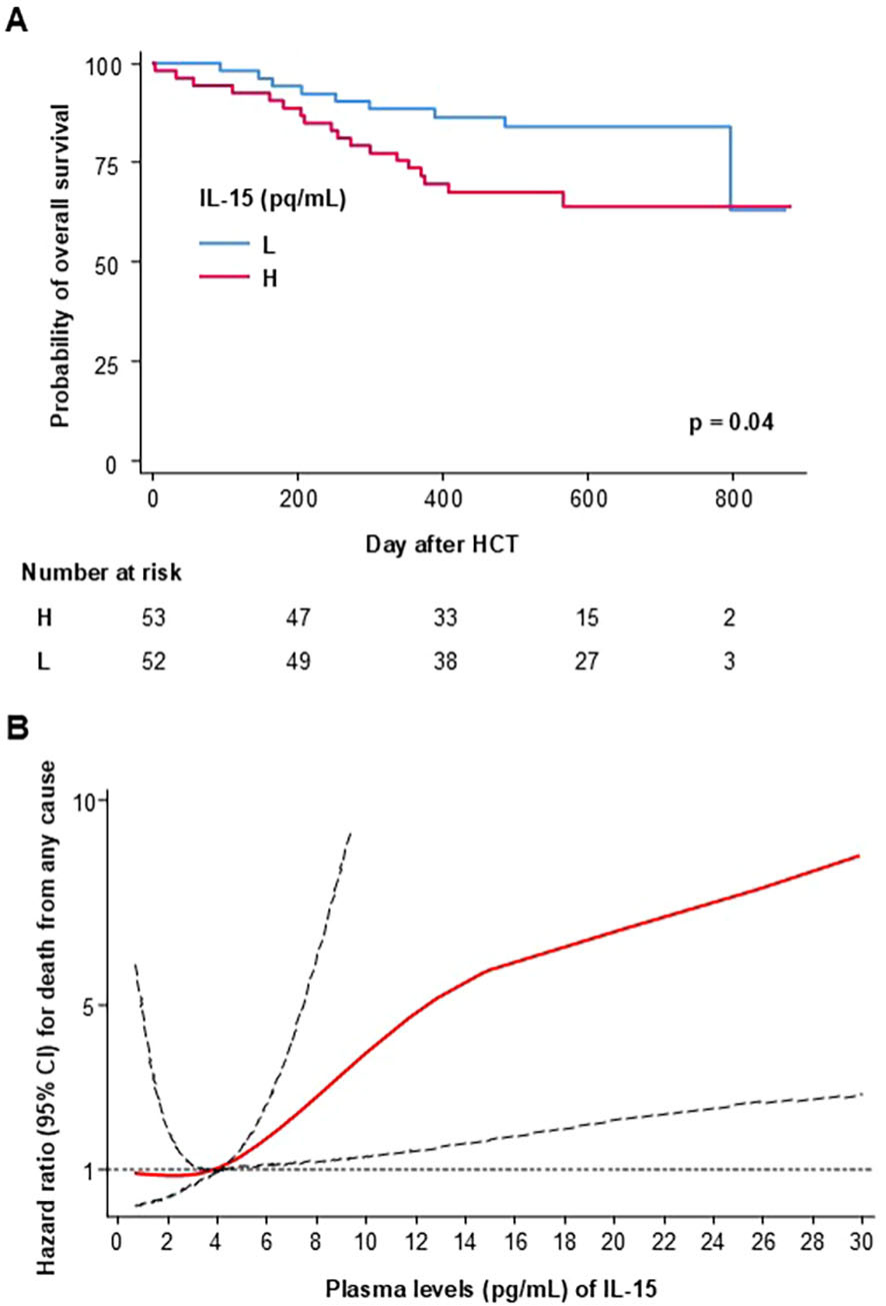
Kaplan-Meier estimated overall survival in patients with high (H) vs. low (L) concentrations of IL-15 on day 28 after HCT, n=105, p=0.039 **(A)**; Risk of death from any cause as a function of IL-15 concentration with solid red lines representing multivariable adjusted hazard ratios and dashed black lines denoting 95% confidence intervals. The median IL-15 concentration (4.1 pg/mL) was set as the reference for the model **(B)**.

**Figure 4:**
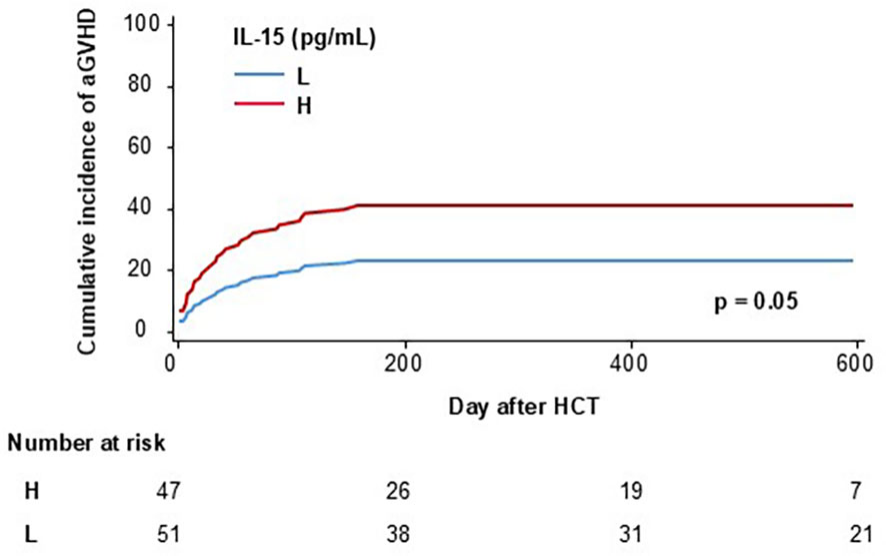
Cumulative incidence of acute GVHD in patients with high (H) vs. low (L) IL-15 concentration day 28 after HCT, *n* = 98, *p* = 0.05. Patients diagnosed with acute GVHD before day 28 were excluded.

In survival analyses, high concentrations compared with low concentrations of IL-15 were significantly associated with reduced OS (*p* = 0.039, **Figure 3A**). When cytokine concentrations were included as continuous variables, increasing IL-15 concentrations were associated with reduced OS with a hazard ratio (HR) of 1.7 (95% CI 1.2–2.5, *p* = 0.004), and reduced RFS with an HR of 1.5 (95% CI 1.1–2.1, *p* = 0.01) in multivariable adjusted models including donor age, HLA match, ATG use, donor type (MUD vs. MRD), sex mismatch (female to male vs. other), and conditioning regimen (MA vs. NMA) (**Table 3**). **Figure 3B** illustrates the change in HRs and 95% confidence intervals for risk of death from any cause as a function of plasma IL-15 concentrations on a continuous scale. The median IL-15 concentration is set as the reference value, i.e., where the HR is set to 1.0, and smoothed curves that fit the data best are shown. **Figure 3B** shows increasing risk of death with increasing IL-15 concentrations, and the same pattern was seen for RFS (data not shown). No association was found when investigating IL-15 and cumulative incidence of death from relapse (*p* = 0.9). When investigating the incidence of aGVHD, high concentrations compared with low concentrations of IL-15 were associated with excess aGVHD (*p* = 0.05) (**Figure 4**). When adjusting the analysis for donor age, HLA match, ATG use, donor type (MUD vs. MRD), sex mismatch (female to male vs. other), and conditioning regimen (MA vs. NMA), results were similar (*p* = 0.04).

**Table 3:** Multivariable adjusted Cox regression analyses on overall survival and relapse-free survival.

Exposure and outcome	Above vs. below median value of IL-15, multivariable adjusted		Log-transformed continuous IL-15 concentration, multivariable adjusted	
Overall survival	HR (95% CI)	*P*-value	HR (95% CI)	*P*-value
IL-15	2.0 (0.9–4.4)	0.1	1.7 (1.2–2.5)	0.004
Donor age (above 30 years vs. below)	2.2 (0.89–5.3)	0.09	2.2 (0.88–5.4)	0.09
HLA-match (other vs. 9-10/10)	2.2 (0.75–6.3)	0.2	1.5 (0.48–4.5)	0.5
ATG (ATG use vs. no use)	1.0 (0.17–5.7)	1.0	1.3 (0.22–7.5)	0.8
Donor type (MUD vs. MRD)	2.0 (0.59–6.8)	0.3	1.6 (0.47–5.4)	0.5
Sex mismatch (female–male vs. other)	3.2 (1.3–7.9)	0.01	2.6 (1.1–6.6)	0.04
Conditioning (MA vs. NMA)	0.58 (0.22–1.5)	0.3	0.54 (0.20–1.5)	0.2
Relapse-free survival				
IL-15	1.4 (0.70–2.6)	0.4	1.5 (1.1–2.1)	0.01
Donor age (above 30 years vs. below)	2.3 (1.1–4.8)	0.04	2.3 (1.1–5.0)	0.03
HLA-match (other vs. 9-10/10)	2.0 (0.79–5.0)	0.14	1.5 (0.60–4.0)	0.4
ATG (ATG use vs. no use)	1.9 (0.56–6.4)	0.3	2.2 (0.64–7.7)	0.2
Donor type (MUD vs. MRD)	1.2 (0.48–3.1)	0.7	1.1 (0.42–2.7)	0.9
Sex mismatch (female–male vs. other)	2.8 (1.2–6.7)	0.02	2.6 (1.1–6.2)	0.03
Conditioning (MA vs. NMA)	0.57 (0.25–1.3)	0.2	0.56 (0.24–1.3)	0.2

Cytokine concentrations are dichotomized by the median value (4.1 pg/mL) or presented on a continuous scale.

IL-7 concentrations were not statistically associated with OS, RFS, or aGVHD (data not shown).

### Immune reconstitution of innate effector cells and clinical outcomes

When investigating innate effector cells, high concentrations compared with low concentrations of γδ T cells were significantly associated with improved OS (*p* = 0.004) and less aGVHD (*p* = 0.02). High concentrations compared with low concentrations of NK cells were significantly associated with improved OS (*p* = 0.03), and in the CD56^bright^ NK cell compartment, high concentrations compared with low concentrations were significantly associated with improved OS (*p* < 0.001), RFS (*p* = 0.005), and a trend towards less aGVHD (*p* = 0.06). These results are in accordance with our previous findings (38, 39), as expected, due to great overlap between the current patient cohort and previous cohorts. All associations between day 28 innate effector cells and clinical outcomes are presented in **Supplementary Table S7**.

## Discussion

In this study of 105 patients, we characterized IL-15 and IL-7 concentrations during immune reconstitution after HCT and examined their correlations to NK cells and γδ T cells. This is, to our knowledge, the first study to examine the relationship between subtypes and activation markers of γδ T cells and NK cells and these specific cytokines after HCT. We furthermore investigated associations between IL-15 and IL-7 and clinical outcomes. IL-15 and IL-7 are key homeostatic cytokines during immune reconstitution after HCT (51, 52). IL-15 is specifically known to affect not only NK cells (53) but also γδ T cells (35, 52), whereas IL-7 primarily regulates overall T-cell proliferation, activation, and homeostasis (51). IL-7 also plays important roles in the early commitment of NK cells from human CD34^+^ hematopoietic progenitor cells, while IL-15 drives functional maturation of NK cells (32). The expression of the receptor CD122 enables NK cells to respond to IL-15, and it has been shown that different NK cell subtypes in relation to their CD56 expression show intrinsically different patterns of responsiveness to IL-15 (54, 55). Similar to NK cells, IL-15 signals via CD122 and the JAK/STAT pathway in γδ T cells, suggesting a common pathway for IL-15 signaling in both innate effector cells (35). IL-15 is predominantly produced by not only monocytes, macrophages, dendritic cells, myeloid cells, and some early hematopoietic cells, but also non-hematopoietic cells such as epithelial cells, fibroblasts, nerve cells, skeletal muscle, and keratinocytes (56). This yields a widespread expression of IL-15 mRNA, which is however regulated at transcriptional and translational checkpoints during steady state (57). Various stress responses are known to induce IL-15 release, e.g., through production of IFN-γ from activated macrophages during inflammation (58). Because of these above-mentioned relations, and as this study is based on a general interest in γδ T-cell and NK cell biology, we chose to focus this study on IL-7 and IL-15 in the post-transplant milieu.

In this study, IL-15 and, to some extent, IL-7 showed the highest concentrations early after HCT and declined over time. Generally, IL-15 was negatively correlated with T-cell concentrations including γδ T cells, NK cells including subtypes, and the activating receptor NKG2D. IL-7 was negatively correlated with T cells and, to a lesser extent, γδ T cells, with the strongest correlations close to HCT. The correlations of IL-15 with innate effector cells tended to be stronger the closer to HCT where especially NK cell concentrations are known to rise rapidly; the correlations with other T cells tended to strengthen until day 91 after HCT and then become non-significant on day 360 after HCT.

Kielsen et al. (30) found elevated IL-15 and IL-7 early post-transplant to be associated with the degree of lymphopenia caused by a myeloablative conditioning regimen combined with ATG treatment. In line with this, we found that patients receiving ATG had a significantly higher IL-7 concentration on day 28 after HCT, consistent with low T-cell number and limited IL-7 consumption. IL-15 and IL-7 availability after HCT may depend on several factors. Previous studies have found high cytokine concentrations in the first weeks after HCT, and during engraftment (29, 30, 59, 60), probably due to systemic inflammation and high concentrations of pro-inflammatory cytokines such as IL-6 and IFN-γ (61), and perhaps further intensified during infectious complications. This is supported by previous studies correlating IL-15 to CRP concentrations 14 days after HCT (29, 30). Of note, when interpreting cytokine data, it should be kept in mind that patients undergoing allogeneic HCT are in a constant state of systemic inflammation, largely due to the procedure itself. Hypothetically, as immune reconstitution follows, repopulating cells may consume cytokines to expand, with higher cell numbers resulting in lower cytokine concentrations. This concept, however, is challenged by the fact that early repopulating cells, especially monocytes, neutrophils, NK cells, and γδ T cells, secrete cytokines themselves (26). In addition, as infectious complications and inflammatory stress in the post-transplant phase might affect especially IL-15 concentrations, it is difficult to clarify whether IL-15 acts as a mechanistic mediator or a surrogate marker of early inflammation. Notably, IL-15 and IL-7 did not correlate at any time point, reflecting a higher level of complexity in the system of cells and cytokines than outlined in this paper.

We found high IL-15 concentration early after HCT to be significantly associated with reduced OS and reduced RFS. Previous studies investigating IL-15 and outcomes in HCT are sparse. Thiant et al. found below-median concentrations of IL-15 14 days after HCT to be associated with increased risk of relapse (59), but without accounting for competing risk of death. In our study, high IL-15 concentrations 28 days after HCT were significantly associated with excess aGVHD. Likewise, Thiant et al. found below-median concentrations of IL-15 30 days after HCT to be associated with less aGVHD (29). In contrast, Kielsen et al. found high concentrations of IL-15 14 days after HCT to be associated with less aGVHD, but only in patients receiving ATG (30). In our cohort, only few patients were treated with ATG. The protective effects of early high NK and γδ T-cell concentrations on post-transplant outcomes were in overall accordance with our previous findings (38, 39).

The association between IL-15 and clinical outcomes together with the correlations between IL-15 and both γδ T cells and NK cells including activated subtypes early after HCT may point at specific mechanistic effects of IL-15 in both innate effector cells. Graft manipulation in haploidentical HCT with αβ T-cell and B-cell depletion offers a setting to investigate the effects of γδ T cells and NK cells early after HCT, as infection control and possibly also anti-leukemic properties rely on these cells due to delayed reconstitution of conventional T cells. A previous study involving 80 pediatric patients found a high engraftment rate, a lower incidence of both acute and chronic GVHD, and a cumulative incidence of relapse comparable to standard HCT (62), indicating both protective GVHD effects and possible early GVL effects of γδ T cells and NK cells. Comparable results have been demonstrated in an adult setting (63). This is in line with our findings of improved survival and less aGVHD with early robust reconstitution of NK cells and γδ T cells. Even in a T-cell-replete setting, there may be essential early effector functions of these cells followed by conventional T cells at later time points, and this might be supported by stronger correlations between IL-15 and the latter further away from HCT.

IL-15 therapy has been intensively studied during the last 10 years. *Ex vivo*, NK cells or γδ T cells incubated with IL-15 show higher proliferative and cytotoxic capacity (64, 65). *In vivo*, recombinant human IL-15 (rhIL-15) monotherapy induced robust expansion of NK cells, γδ T cells, and CD8^+^ T cells, but was not associated with any objective responses in solid tumors (66, 67). Cooley et al. investigated rhIL-15 therapy in patients with relapsed and/or refractory acute myeloid leukemia (AML) following lymphodepletion and haploidentical NK cell infusion. rhIL-15 therapy induced clinical remission in 32% and 40% of patients; however, persistence of NK cells on day 14 was only seen in some of the patients, which could be explained by the concomitant stimulation of recipient CD8^+^ T cells, which may reject donor NK cells (68), as supported by another recent study using an IL-15 receptor agonist (ALT-803) (69). The first-in-human trial of ALT-803 examined patients relapsing > 60 days after HCT and found treatment to be well tolerated, with no dose-limiting toxicities and no CRS or GVHD. ALT-803 stimulated the activation, proliferation, and expansion of NK cells and CD8^+^ T cells without increasing regulatory T cells. In this study, CD56^bright^ NK cells showed the highest proliferation, and interestingly, treatment yielded upregulation of the activating receptor NKG2D (70). This is supported by Wagner et al., where CD56^bright^ NK cells, when primed with IL-15, showed enhanced cytotoxicity, degranulation, and cytokine production both *in vitro* and *in vivo* (71). These data point at IL-15 being a promising treatment option, especially in combination with NK cells or γδ T cells; however, route, dose/interval, and formulation are important factors to consider (34). Furthermore, the risk of cytokine competition between CD8^+^ T cells and innate effector cells, with potential impaired NK cell reconstitution, should be acknowledged in a T-cell-replete setting.

The overall strength of this study is the prospective design with characterizations of both cell concentration and cytokines in a large cohort of patients treated with HCT at a single center, and with available clinical data on all patients at the end of follow-up. Limitations of this study are the relatively heterogeneous patient population regarding patient age, underlying hematologic diagnoses, different conditioning regimens, ATG use, donor type, and graft source. This may introduce variability in immune reconstitution and cytokine dynamics, which complicates interpretation. As cytokine levels are highly influenced by infections and inflammation, information on infections around day 28 would have added value to this study; however, this information was not available. Furthermore, functional studies of cell subsets and cytokine release could have contributed to the mechanistic understanding of the observed results and especially aided in the interpretation of the inverse associations between cells and cytokines but were not in the scope of this study. Regarding methodological limitations, we cannot exclude the idea that sample freezing might have affected cytokine stability or assay sensitivity. In particular, freeze–thaw cycles have been shown to affect cytokine concentrations (72); however, in this study, we only did one additional freeze–thaw cycle before analyses. A recent study involving 9,872 Danish blood donors investigated the effect of storage time for IL-15 and IL-7 analyses and found no significant change in concentrations for both cytokines for each assay per storage year (73).

In conclusion, in this study of 105 patients, high IL-15 measured early after HCT was significantly associated with reduced OS, reduced RFS, and excess aGVHD. Furthermore, IL-15 was inversely correlated with early robust reconstitution of NK cells and γδ T cells, which remained associated with improved clinical outcomes after HCT. Our findings might support the concept of cytokine consumption during the expansion of NK cells and γδ T cells and may indicate specific mechanistic effects of IL-15 in these innate effector cells during early immune reconstitution after HCT. These results warrant further investigations of IL-15 therapy in combination with innate effector cells.

## Data Availability

The datasets presented in this article are readily available, if the request is in accordance with national Danish legislation. Requests to access the datasets should be directed to marie.warny@regionh.dk?
