# Supplementary material for: Interleukin-15 and innate effector cells as predictors of outcome in allogeneic hematopoietic cell transplantation
Source: Front Immunol. 2026 Feb 2;16:1649313. doi: 10.3389/fimmu.2025.1649313 (PMC12907447; doi:10.3389/fimmu.2025.1649313)
Supplement: Supplementary file 1 [file DataSheet1.docx]

**Interleukin-15 and innate effector cells as predictors of outcome in allogeneic hematopoietic cell transplantation**

**Authors:** Marie Warny, Sisse Rye Ostrowski, Søren Lykke Petersen, Lone Smidstrup Friis, Brian Thomas Kornblit, Niels Smedegaard Andersen, Ida Schjødt, Margit Hørup Larsen, Janne Amstrup Møller, Eva Kannik Haastrup, Henrik Sengeløv, and Lia Minculescu

**Supplementary material**

Table of contents Page No.

**Supplementary Table S1:** Panels for leukocyte phenotyping 2

**Supplementary Figure S1:** Gating strategies 3

**Supplementary Table S2:** Subset definitions and phenotypes 5

**Supplementary Table S3:** Univariate analyses of pre-transplant factors and impact on

overall survival and relapse-free survival after HCT 6

**Supplementary Table S4:** Concentrations (pg/mL) of IL-15 (upper panel)

and IL-7 (lower panel) during the first year after HCT 7

**Supplementary Table S5**: Correlations between cytokine concentrations and cell subtypes

day 28, 56, 91, 181, and 365 after HCT 8

**Supplementary Table S6**: Absolute cell concentrations in patients and donors 11

**Supplementary Table S7**: Associations between day 28 lymphocyte counts and

clinical outcomes 12

**Supplementary Table S1:** Two-tube panel of monoclonal antibodies (mAB) used for leukocyte phenotyping. Antibodies were titrated and used in saturating concentrations.

| **Fluorochrome** | **mAB** | **Clone** | **Manufacturer catalog no.** |
| --- | --- | --- | --- |
| **Tube 1** | | | |
| FITC | TCRαβ | Clone WT31 | BD-333140 |
| PE | TCRγδ | Clone 11F2 | BD-333141 |
| PerCp Cy5.5 | CD4 | Clone SK3 | BD-332772 |
| PE-Cy7 | CD45RA | Clone L48 | BD-337186 |
| Alexa Fluor 647 | CD197 | Clone 150503 | BD-560816 |
| - |  |  |  |
| APC-H7 | CD45RO | Clone UCHL1 | BD-561137 |
| V450 | HLA-DR | Clone L243 | BD-655874 |
| V500 | CD3 | Clone SP34-2 | BD-560770 |
| BV605 | CD8 | Clone SK1 | BD-564116 |
| **Tube 2** | | | |
| FITC | TCRVδ2 | Clone 123R3 | Miltenyi-130-095-798 |
| PE | TCRγδ | Clone 11F2 | BD-333141 |
| - |  |  |  |
| PE-Vio770 | TCRVδ1 | Clone REA173 | Miltenyi-130-100-540 |
| APC | CD314 | Clone 1D11 | BD-558071 |
| - |  |  |  |
| APC-H7 | CD16 | Clone 3G8 | BD-560195 |
| V450 | CD56 | Clone B159 | BD-560360 |
| V500 | CD3 | Clone SP34-2 | BD-560770 |
| BV605 | CD337 | Clone p30-15 | BD-563384 |

**Supplementary Figure S1:** Gating strategies. Extracts from flow panels and gating strategies in tube 1 and tube 2. Dead cells/debris and dublets were remowed as shown in the dot plot forward and side scatter (FSC/SSC) and FSC Area/FSC High. Lymphocytes were identified based on their forward and side scatter properties. In tube 1, CD3pos events were selected on the CD3/SSC dot plot and subsequintly CD3 T cells were separated in a TCR αβ/TCR γδ plot and TCR αβ T cells were further separated in a CD4/CD8 plot (not shown). TCR αβ T cells, TCR γδ T cells and CD4- and CD8 T cells were separately investigated for differentation markers in a CD45RA/CD45RO plot for identification of CD45RAneg/CD45RApos memory cell phenotypes, and a CD45RA/CD197 plot for identification of central(CD45RAnegCD197pos)/effector(CD45RAneg/CD197neg) memory-, CD45RApos/CD197pos naive-, and CD45RApos/CD197neg TEMRA cell phenotypes. The expression of HLA-DR were investigated in histograms for TCR αβ T cells, TCR γδ T cells and CD4- and CD8 T cells separately. In tube 2, TCR γδ (CD3pos) T cells were identified in a CD3/TCR γδ plot, and TCR γδ T cells were furthermore separated in subtypes in a TCR Vδ2/TCR Vδ1 plot. NK cells were identified in a CD56/CD16 plot gated on CD3neg lymphocytes and 3 populations (CD56bright, CD16pos/CD56pos and CD16bright) were identified based on CD16- and CD56 expression. TCR γδ T cells, TCR Vδ1 T cells, TCR Vδ2 T cells, CD56bright NK cells, CD16pos/CD56pos NK cells and CD16bright NK cells were separately investigated for the expression of CD314 and CD337 in histograms and analyzed based on fractions of cells positive as well as the MFI (mean fluorescence intensity) expression.

**Tube 1**


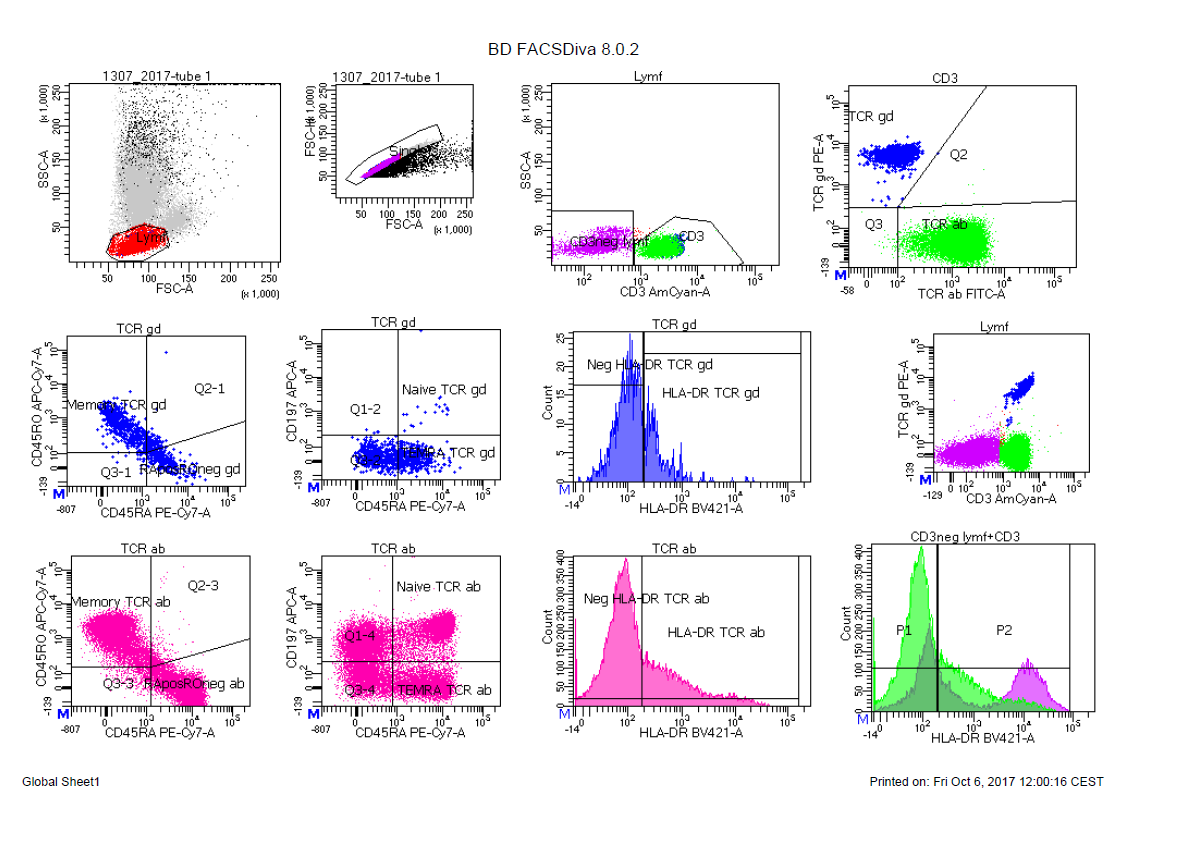

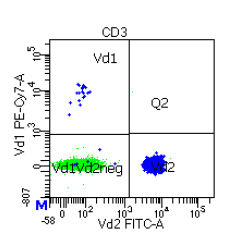

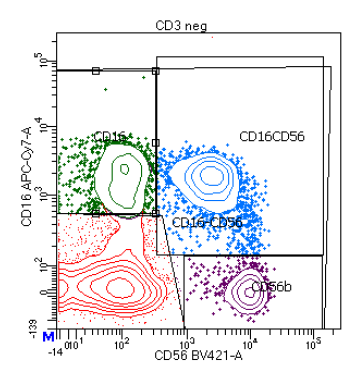


**Tube 2**

**Supplementary table S2:** Subset definitions and phenotypes. The absolute concentrations of CD3, CD4, CD8 and NK cells were calculated by the BD TM Trucount system. Additional fractions and concentrations were calculated from the panels in Supplementary Table S1.

| Subset definition | Phenotype |
| --- | --- |
| CD3+TCR γδ+  CD3+TCR γδ+CD45RA+CD197+  CD3+TCR γδ+CD45RA-CD197+  CD3+TCR γδ+CD45RA-CD197-  CD3+TCR γδ+CD45RA+CD197-  CD3+TCR γδ+Vδ1+  CD3+TCR γδ Vδ2+  CD3+TCR αβ+CD4+  CD3+TCR αβ+ CD4+CD45RA+CD197+  CD3+TCR αβ+ CD4+CD45RA-CD197+  CD3+TCR αβ+ CD4+CD45RA-CD197-  CD3+TCR αβ+ CD4+CD45RA+CD197-  CD3+TCR αβ+CD8+  CD3+TCR αβ+ CD8+CD45RA+CD197+  CD3+TCR αβ+ CD8+CD45RA-CD197+  CD3+TCR αβ+ CD8+CD45RA-CD197-  CD3+TCR αβ+ CD8+CD45RA+CD197-  CD16/CD56+  CD16lowCD56++  CD16+CD56+  CD16++CD56low | TCR γδ cells  Naive TCR γδ cells  Central memory TCR γδ cells  Effector memory TCR γδ cells  TEMRA TCR γδ cells  TCR Vδ1 cells  TCR Vδ2 cells  CD4 T cells  Naive CD4 T cells  Central memory CD4 T cells  Effector memory CD4 T cells  TEMRA CD4 T cells  CD8 T cells  Naive CD8 T cells  Central memory CD8 T cells  Effector memory CD8 T cells  TEMRA CD8 T cells  NK cells  CD56bright NK cells  CD16CD56 NK cells  CD16bright NK cells |
|  |  |
|  |  |

**Supplementary Table S3:** Univariate analyses of pre-transplant factors and impact on overall survival and relapse-free survival after HCT, n=105.

| **Variable** | **n** | **Overall survival** | **Relapse-free survival** |
| --- | --- | --- | --- |
|  |  | HR (95% CI) p-value | HR (95% CI) p-value |
| **Recipient age,**  > 45 years  < 45 years  **Disease type,**  AML/MDS  Other  **Donor age,**  >30 years  <30 years  **Donor type,**  Matched unrelated donor  Matched related donor  **Graft source,**  BM  PBSC  **Conditioning regimen,**  Myeloablative  Non-myeloablative  **ATG,**  No  Yes  **Disease Risk Index*,**  High  Intermediate  Low  **HLA-match,**  9 – 10/10 allele match  Other  **Sex Mismatch,**  Other  Female-male | 81  24  69  36  60  45  82  23  15  90  48  57  93  12  11  84  10  94  11  94  11 | 1.00  0.77 (0.3-2.0) 0.6  1.00  1.40 (0.6-3.0) 0.4  1.00  0.46 (0.2-1.0) 0.05  1.00  0.66 (0.2-1.9) 0.4  1.00  1.03 (0.4-3.0) 1.0  1.00  2.19 (1.0-5.0) 0.06  1.00  0.68 (0.2-2.9) 0.6    -  1.00  4.17 (1.7-10) 0.001  1.00  3.49 (1.5-8.3) 0.005 | 1.00  0.80 (0.4-1.8) 0.6  1.00  1.28 (0.7-2.5) 0.5  1.00  0.39 (0.2-0.8) 0.005  1.00  1.17 (0.5-2.6) 0.7  1.00  1.14 (0.4-2.9) 0.8  1.00  1.70 (0.9-3.3) 0.1  1.00  1.45 (0.6-3.7) 0.5  1.00  1.15 (0.4-3.3) 0.8  0.23 (0.03-2.1) 0.2  1.00  3.20 (1.5-7.1) 0.004  1.00  2.55 (1.1-5.8) 0.03 |

*Disease Risk Index: as there were no deaths in the low-risk group during the observation time, no estimate of this variable is included for overall survival. AML; acute myeloid leukemia, MDS; myelodysplastic syndrome, BM; bone marrow, PBSC; peripheral blood stem cells, ATG; anti-thymocyte-globulin.

**Supplementary Table S4** Concentrations (pg/mL) of IL-15 (upper panel) and IL-7 (lower panel) during the first year after HCT.

| **IL-15 (pg/mL)** | | | | | | |
| --- | --- | --- | --- | --- | --- | --- |
|  | | **Day 28** | **Day 56** | **Day 91** | **Day 180** | **Day 365** |
| **N** |  | 105 | 102 | 98 | 85 | 65 |
| **Median** | | 4.1 | 2.0 | 2.1 | 1.9 | 1.5 |
| **Minimum** | | 0.7 | 0.5 | 0.3 | 0.3 | 0.3 |
| **Maximum** | | 43.8 | 21.3 | 26.2 | 16.6 | 13.1 |
| **Percentiles** | **25^th^** | 2.6 | 1.4 | 1.3 | 1.2 | 0.9 |
|  | **75^th^** | 6.3 | 3.1 | 3.6 | 2.5 | 2.4 |

| **IL-7 (pg/mL)** | | | | | | |
| --- | --- | --- | --- | --- | --- | --- |
|  | | **Day 28** | **Day 56** | **Day 91** | **Day 180** | **Day 365** |
| **N** |  | 105 | 102 | 98 | 83 | 65 |
| **Median** | | 3.1 | 3.6 | 2.9 | 2.4 | 3.8 |
| **Minimum** | | 0.2 | 0.6 | 0.3 | 0.3 | 0.9 |
| **Maximum** | | 42.3 | 27.7 | 16.7 | 13.7 | 26.7 |
| **Percentiles** | **25^th^** | 1.3 | 2.0 | 2.0 | 1.6 | 2.5 |
|  | **75^th^** | 5.7 | 5.4 | 5.0 | 4.3 | 6.0 |

**Supplementary Table S5** Correlations between cytokine concentrations and cell subtypes day 28, 56, 91, 181, and 365 after HCT. IL-15 (upper panel) and IL-7 (lower panel).

| **IL-15** |  |  |  | |  |  | |  |  |  |  |  |  |  |  |  |
| --- | --- | --- | --- | --- | --- | --- | --- | --- | --- | --- | --- | --- | --- | --- | --- | --- |
| **Day after HSCT** | **28** | |  | | **56** | | |  | **91** | |  | **180** | |  | **365** | |
| **Number of patients** | **105** | |  | | **102** | | |  | **98** | |  | **85** | |  | **65** | |
| **Correlations** | **Coefficient** | **p-value** | |  | **Coefficient** | **p-value** | |  | **Coefficient** | **p-value** |  | **Coefficient** | **p-value** |  | **Coefficient** | **p-value** |
|  |  |  |  | |  |  | |  |  |  |  |  |  |  |  |  |
| **NK cells** | -0.497 | <0.001 |  | | -0.524 | <0.001 | |  | -0.438 | <0.001 |  | -0.229 | 0.04 |  | -0.065 | 0.6 |
| CD56^dim^ (CD16CD56) | -0.568 | <0.001 |  | | -0.574 | <0.001 | |  | -0.459 | <0.001 |  | -0.181 | 0.1 |  | -0.039 | 0.8 |
| CD56^bright^ | -0.474 | <0.001 |  | | -0.392 | <0.001 | |  | -0.223 | 0.03 |  | -0.236 | 0.03 |  | -0.216 | 0.09 |
| NKG2D* |  |  |  | |  |  | |  |  |  |  |  |  |  |  |  |
| CD16CD314 | -0.419 | <0.001 |  | | -0.531 | <0.001 | |  | -0.557 | <0.001 |  | -0.462 | <0.001 |  | -0.350 | 0.005 |
| CD16CD56CD314 | -0.563 | <0.001 |  | | -0.576 | <0.001 | |  | -0.463 | <0.001 |  | -0.201 | 0.07 |  | -0.036 | 0.8 |
| CD56bCD314 | -0.470 | <0.001 |  | | -0.391 | <0.001 | |  | -0.216 | 0.03 |  | -0.225 | 0.04 |  | -0.224 | 0.08 |
|  |  |  |  | |  |  | |  |  |  |  |  |  |  |  |  |
| **γδ T cells** | -0.341 | <0.001 |  | | -0.317 | 0.001 | |  | -0.338 | <0.001 |  | -0.273 | 0.01 |  | -0.258 | 0.04 |
| Vδ1 | -0.252 | 0.01 |  | | -0.339 | <0.001 | |  | -0.284 | 0.005 |  | -0.192 | 0.08 |  | -0.237 | 0.06 |
| Vδ2 | -0.388 | <0.001 |  | | -0.303 | 0.002 | |  | -0.352 | <0.001 |  | -0.318 | 0.003 |  | -0.157 | 0.2 |
| NKG2D* |  |  |  | |  |  | |  |  |  |  |  |  |  |  |  |
| Vδ1CD314 | -0.194 | 0.05 |  | | -0.325 | <0.001 | |  | -0.266 | 0.009 |  | -0.178 | 0.1 |  | -0.246 | 0.05 |
| Vδ2CD314 | -0.389 | <0.001 |  | | -0.305 | 0.002 | |  | -0.350 | <0.001 |  | -0.315 | 0.004 |  | -0.154 | 0.2 |
| γδCD314 | -0.349 | <0.001 |  | | -0.318 | 0.001 | |  | -0.335 | <0.001 |  | -0.283 | 0.009 |  | -0.251 | 0.05 |
| HLA-DR* | -0.305 | 0.002 |  | | -0.250 | 0.01 | |  | -0.346 | <0.001 |  | -0.263 | 0.02 |  | -0.256 | 0.04 |
| TCRγδParent* | -0.281 | 0.004 |  | | -0.185 | 0.06 | |  | -0.121 | 0.2 |  | -0.060 | 0.6 |  | -0.187 | 0.1 |
|  |  |  |  | |  |  | |  |  |  |  |  |  |  |  |  |
| **CD3 T cells** | -0.243 | 0.01 |  | | -0.239 | 0.02 | |  | -0.384 | <0.001 |  | -0.350 | 0.001 |  | -0.247 | 0.05 |
|  |  |  |  | |  |  | |  |  |  |  |  |  |  |  |  |
| **CD4 T cells** | -0.280 | 0.004 |  | | -0.308 | 0.002 | |  | -0.385 | <0.001 |  | -0.294 | 0.006 |  | -0.212 | 0.09 |
| HLA-DR* | -0.171 | 0.09 |  | | -0.229 | 0.02 | |  | -0.433 | <0.001 |  | -0.411 | <0.001 |  | -0.158 | 0.2 |
|  |  |  |  | |  |  | |  |  |  |  |  |  |  |  |  |
| **CD8 T cells** | -0.220 | 0.02 |  | | -0.150 | 0.1 | |  | -0.312 | 0.002 |  | -0.311 | 0.004 |  | -0.237 | 0.06 |
| HLA-DR* | -0.171 | 0.08 |  | | -0.056 | 0.6 | |  | -0.291 | 0.004 |  | -0.289 | 0.007 |  | -0.198 | 0.1 |
| **IL-7** |  |  | |  |  |  |  | |  |  |  |  |  |  |  |  |
| **Day after HSCT** | **28** | | |  | **56** | |  | | **91** | |  | **180** | |  | **365** | |
| **Number of patients** | **105** | | |  | **102** | |  | | **98** | |  | **85** | |  | **65** | |
| **Correlations** | **Coefficient** | **p-value** | |  | **Coefficient** | **p-value** | |  | **Coefficient** | **p-value** |  | **Coefficient** | **p-value** |  | **Coefficient** | **p-value** |
|  |  |  | |  |  |  |  | |  |  |  |  |  |  |  |  |
| **NK cells** | 0.033 | 0.7 | |  | 0.086 | 0.4 |  | | 0.136 | 0.2 |  | -0.110 | 0.9 |  | 0.115 | 0.4 |
| CD56^dim^ (CD16CD56) | -0.049 | 0.6 | |  | 0.047 | 0.6 |  | | 0.064 | 0.5 |  | -0.062 | 0.6 |  | 0.103 | 0.4 |
| CD56^bright^ | 0.175 | 0.07 | |  | 0.163 | 0.1 |  | | 0.175 | 0.09 |  | 0.232 | 0.04 |  | 0.097 | 0.4 |
| NKG2D* |  |  | |  |  |  |  | |  |  |  |  |  |  |  |  |
| CD16CD314 | 0.132 | 0.2 | |  | 0.133 | 0.2 |  | | 0.144 | 0.2 |  | 0.035 | 0.8 |  | -0.096 | 0.4 |
| CD16CD56CD314 | -0.042 | 0.7 | |  | 0.044 | 0.7 |  | | 0.057 | 0.6 |  | -0.063 | 0.6 |  | 0.106 | 0.4 |
| CD56bCD314 | 0.184 | 0.06 | |  | 0.164 | 0.1 |  | | 0.178 | 0.08 |  | 0.223 | 0.04 |  | 0.072 | 0.6 |
|  |  |  | |  |  |  |  | |  |  |  |  |  |  |  |  |
| **γδ** | -0.266 | 0.006 | |  | -0.047 | 0.6 |  | | -0.010 | 0.9 |  | -0.213 | 0.05 |  | -0.206 | 0.1 |
| Vδ1 | -0.108 | 0.3 | |  | 0.101 | 0.3 |  | | 0.183 | 0.08 |  | -0.144 | 0.2 |  | -0.109 | 0.4 |
| Vδ2 | -0.303 | 0.002 | |  | -0.083 | 0.4 |  | | -0.073 | 0.5 |  | -0.151 | 0.2 |  | -0.104 | 0.4 |
| NKG2D* |  |  | |  |  |  |  | |  |  |  |  |  |  |  |  |
| Vδ1CD314 | -0.106 | 0.3 | |  | 0.126 | 0.2 |  | | 0.205 | 0.05 |  | -0.151 | 0.2 |  | -0.111 | 0.4 |
| Vδ2CD314 | -0.314 | 0.001 | |  | -0.085 | 0.4 |  | | -0.073 | 0.5 |  | -0.151 | 0.2 |  | -0.106 | 0.4 |
| γδCD314 | -0.236 | 0.02 | |  | -0.049 | 0.6 |  | | -0.010 | 0.9 |  | -0.222 | 0.05 |  | -0.212 | 0.09 |
| HLA-DR* | -0.184 | 0.06 | |  | 0.019 | 0.8 |  | | -0.030 | 0.8 |  | -0.251 | 0.02 |  | -0.233 | 0.06 |
| TCRγδParent* | 0.099 | 0.3 | |  | 0.164 | 0.1 |  | | 0.168 | 0.1 |  | -0.067 | 0.5 |  | -0.188 | 0.1 |
|  |  |  | |  |  |  |  | |  |  |  |  |  |  |  |  |
| **CD3 T cells** | -0.471 | <0.001 | |  | -0.274 | 0.005 |  | | -0.210 | 0.04 |  | -0.219 | 0.05 |  | -0.028 | 0.8 |
|  |  |  | |  |  |  |  | |  |  |  |  |  |  |  |  |
| **CD4 T cells** | -0.493 | <0.001 | |  | -0.381 | <0.001 |  | | -0.302 | 0.003 |  | -0.108 | 0.3 |  | 0.030 | 0.8 |
| HLA-DR* | -0.373 | <0.001 | |  | -0.124 | 0.2 |  | | -0.172 | 0.09 |  | -0.038 | 0.7 |  | 0.023 | 0.9 |
|  |  |  | |  |  |  |  | |  |  |  |  |  |  |  |  |
| **CD8 T cells** | -0.415 | <0.001 | |  | -0.175 | 0.08 |  | | -0.125 | 0.2 |  | -0.211 | 0.06 |  | -0.052 | 0.7 |
| HLA-DR* | -0.188 | 0.05 | |  | -0.013 | 0.9 |  | | -0.069 | 0.5 |  | -0.170 | 0.1 |  | -0.064 | 0.6 |

***** HLA-DR is a marker of activation on γδ T cells, CD4^+^ T cells, and CD8^+^ T cells. NKG2D is an activating receptor on γδ T cells, (γδCD314), Vδ1 cells (Vδ1CD314), Vδ2 cells (Vδ2CD314), CD56^dim^ NK cells (CD16CD56CD314), and CD56^bright^ NK cells (CD56bCD314). TCRγδParent is the fraction of γδ T cells of all CD3 cells.

**Supplementary Table S6:** Absolute cell concentrations in patients and donors. NA: not available.

| Concentration of subset, 10^6^/L,  median (IQR) | **Day 28**  N = 105 | **Day 56**  N = 102 | **Day 91**  N = 98 | **Day 180**  N = 85 | **Day 365**  N = 65 | **Healthy donors**  N=53 |
| --- | --- | --- | --- | --- | --- | --- |
| CD3 | 420 (230-550) | 420 (260-740) | 460 (240-750) | 860 (470-1,400) | 1,200 (730-2,000) | 1,500 (1,150-1,700) |
| CD4 | 190 (110-300) | 185 (110-280) | 210 (100-300) | 280 (170-420) | 410 (250-490) | 930 (715-1,100) |
| CD8 | 150 (71-240) | 175 (100-380) | 240 (100-440) | 450 (210-950) | 650 (370-1,400) | 345 (430-630) |
| δγ | 18 (6.5-44) | 21 (7.1-50) | 21 (6.6-53) | 37 (12-86) | 65 (19-110) | 41 (25-81) |
| Vδ1 | 1.5 (0.75-3.4) | 1.6 (0.61-4.7) | 1.6 (0.60-6.8) | 4.4 (1.6-23) | 7.6 (3.3-35) | NA |
| Vδ2 | 12 (4.8-42) | 17 (4.9-39) | 14 (4.0-38) | 15 (6.2-54) | 25 (7.2-62) | NA |
| NK | 280 (140-380) | 190 (110-330) | 160 (100-270) | 240 (130-380) | 230 (150-305) | 200 (140-300) |
| CD56^dim^ | 157 (82.4-256) | 113 (70.1-198) | 106 (64.6-178) | 145 (97.0-281) | 175 (112-286) | 165 (113-250) |
| CD56^bright^ | 57 (27-94) | 31 (17-59) | 23 (14-38) | 17 (12-32) | 17 (9.8-26) | 14 (8.9-20) |

**Supplementary Table S7:** Associations between day 28 lymphocyte counts and clinical outcomes.

| **γδ T cells** |  |  |  |
| --- | --- | --- | --- |
|  | **p (Log-rank)** | **HR (Cox)** | **p(Cox)** |
| OS* | 0.004 | 0.9 (0.9-1.0) | 0.006 |
| RFS* | 0.1 | 0.9 (0.9-1.0) | 0.04 |
|  | **p (CI)** | **HR (Fine&Gray)** | **p(Fine&Gray)** |
| Relapse | 0.5 | 0.9 (0.9-1.0) | 0.1 |
| aGVHD | 0.02 | 0.9 (0.9-1.0) | 0.4 |
|  |  |  |  |
| **Vδ1** |  |  |  |
|  | **p (Log-rank)** | **HR (Cox)** | **p(Cox)** |
| OS* | 0.2 | 1.0 (0.9-1.0) | 0.2 |
| RFS* | 0.3 | 1.0 (0.9-1.0) | 0.4 |
|  | **p (CI)** | **HR (Fine&Gray)** | **p(Fine&Gray)** |
| Relapse | 0.6 | 1.0 (0.9-1.0) | 0.3 |
| aGVHD | 0.07 | 1.0 (0.9-1.1) | 0.7 |
|  |  |  |  |
| **Vδ2** |  |  |  |
|  | **p (Log-rank)** | **HR (Cox)** | **p(Cox)** |
| OS* | 0.001 | 0.9 (0.8-0.9) | <0.001 |
| RFS* | 0.02 | 0.9 (0.9-1.0) | 0.001 |
|  | **p (CI)** | **HR (Fine&Gray)** | **p(Fine&Gray)** |
| Relapse | 0.2 | 1.0 (0.9-1.0) | 0.2 |
| aGVHD | 0.02 | 1.0 (0.9-1.0) | 0.7 |
|  |  |  |  |
| **NK cells** |  |  |  |
|  | **p (Log-rank)** | **HR (Cox)** | **p(Cox)** |
| OS* | 0.03 | 0.6 (0.4-0.8) | <0.001 |
| RFS* | 0.06 | 0.7 (0.5-0.9) | 0.003 |
|  | **p (CI)** | **HR (Fine&Gray)** | **p(Fine&Gray)** |
| Relapse | 0.5 | 0.8 (0.6-1.1) | 0.2 |
| aGVHD | 0.1 | 0.7 (0.5-0.9) | 0.01 |
|  |  |  |  |
| **CD56^dim^** |  |  |  |
|  | **p (Log-rank)** | **HR (Cox)** | **p(Cox)** |
| OS* | 0.1 | 0.7 (0.5-0.8) | 0.001 |
| RFS* | 0.1 | 0.7 (0.6-0.9) | 0.005 |
|  | **p (CI)** | **HR (Fine&Gray)** | **p(Fine&Gray)** |
| Relapse | 0.3 | 0.8 (0.6-1.0) | 0.08 |
| aGVHD | 0.4 | 0.9 (0.7-1.1) | 0.2 |
|  |  |  |  |
| **CD56^bright^** |  |  |  |
|  | **p (Log-rank)** | **HR (Cox)** | **p(Cox)** |
| OS* | <0.001 | 0.5 (0.4-0.7) | <0.001 |
| RFS* | 0.005 | 0.6 (0.5-0.8) | <0.001 |
|  | **p (CI)** | **HR (Fine&Gray)** | **p(Fine&Gray)** |
| Relapse | 0.5 | 0.8 (0.6-1.0) | 0.08 |
| aGVHD | 0.06 | 0.8 (0.6-1.0) | 0.08 |

* OS (overall survival) and RFS (relapse-free survival) were assessed using log-rank test and Cox regression. Risk of relapse and acute GVHD were assessed using competing risk regression with cumulative incidence and Fine and Gray proportional hazard regression, with death as competing event. HR: hazard ratio, CI: cumulative incidence, p: p-value
